# LINC00665 promotes the progression and immune evasion of lung cancer by facilitating the translation of TCF7 protein through dependence on IRES

**DOI:** 10.1186/s12935-024-03411-4

**Published:** 2024-06-29

**Authors:** Chaonan Han, Jinchen Su, Yue Pei, Xiangyu Su, Di Zheng

**Affiliations:** 1 Department of Oncology, Shanghai Pulmonary Hospital, School of Medicine, Tongji University, Shanghai, 200443 China; 2 School of Medicine, Shihezi University, Shihezi, 832000 Xinjiang China; 3 Department of Laboratory Medicine, Yixing Hospital of Traditional Chinese Medicine, No.128 East Yangquan Road, Yicheng Subdistrict, Yixing, 214200 Jiangsu China; 4 Department of Oncology, School of Medicine, Zhongda Hospital, Southeast University, Nanjing, 210009 Jiangsu P.R. China

**Keywords:** Lung cancer, LINC00665, HHLA2, TCF7, NK cells, Immune escape

## Abstract

**Objective:**

To investigate the influence of LINC00665 on the development and immune evasion of lung cancer.

**Methods:**

Tumor tissues and corresponding adjacent tissues were collected from 84 lung cancer patients, categorized into non-metastatic (*n* = 58) and metastatic (*n* = 26) groups. LINC00665 expression in lung cancer and metastatic lung cancer tissues was assessed via qRT-PCR. Pearson correlation analysis was conducted to examine the correlation between LINC00665 and immune-modulating cytokines (TGF-β, IL-10, IL-1β, IFN-γ, IL-2, TNF-α). A549 and H1299 cells, with relatively high LINC00665 expression, were used for in vitro studies. Cells were transfected with LINC00665-targeting shRNA, and changes in proliferation, apoptosis, migration, invasion, and NK cell cytotoxicity were assessed. Downstream molecular mechanisms of LINC00665 were investigated using GEO database analysis, highlighting the association with HHLA2. LINC00665’s role in promoting HHLA2 expression via binding with TCF7 was explored. In low LINC00665-expressing A549/H1299 cells, overexpression of HHLA2 was performed to evaluate effects on malignant behavior and NK cell sensitivity. A xenograft model was established for in vivo validation through tumor volume and weight measurements, Ki-67 immunoreactivity analysis, and flow cytometry analysis of CD107a + NK cells.

**Results:**

LINC00665, TCF7 mRNA, and HHLA2 mRNA expression levels were significantly higher in lung cancer tissues than adjacent tissues, with non-metastatic lung cancer showing higher expression than metastatic lung cancer. In metastatic lung cancer, LINC00665 positively correlated with immune-suppressive cytokines (TGF-β, IL-10, IL-1β) and negatively correlated with anti-tumor cytokines (IFN-γ, IL-2, TNF-α). LINC00665 knockdown significantly inhibited lung cancer cell growth and metastasis, promoting sensitivity to NK cells. Further analysis revealed that LINC00665 recruits transcription factor TCF7 to upregulate HHLA2 expression in lung cancer cells, thereby facilitating lung cancer development and immune escape.

**Conclusion:**

LINC00665, through recruitment of TCF7 and upregulation of HHLA2, inhibits NK cell cytotoxicity, promoting the development and immune evasion of lung cancer.

**Supplementary Information:**

The online version contains supplementary material available at 10.1186/s12935-024-03411-4.

## Introduction

Lung cancer is a malignant tumor with high incidence and mortality rates. According to the 2018 global cancer statistics, lung cancer is the most frequently diagnosed cancer and a leading cause of cancer-related deaths [1]. Within lung cancer, 80–85% is classified as non-small cell lung cancer (NSCLC) [2, 3], and the 5-year survival rate for stage III or IV NSCLC with lymph node metastasis is less than 19% [4]. Despite significant progress in the treatment of lung cancer through radiotherapy, chemotherapy, targeted therapy, and/or immunotherapy, the prognosis remains poor. Immune escape is considered a crucial hallmark of cancer, significantly promoting tumor progression and metastasis. It is increasingly recognized as a determining factor in immunotherapy [5]. Natural killer (NK) cells are a subset of innate lymphoid cells known for their ability to spontaneously kill target cells [6]. They are generally considered a part of type I innate-like cells (ILC1s) and are defined as effector cells similar to cytotoxic T cells. NK cells exert innate cytotoxicity against primary tumor cells and metastases by inhibiting proliferation, migration, and colonization in distant tissues [7]. In addition to their cytotoxic role, NK cells produce a substantial amount of cytokines, primarily interferon-gamma (IFN-γ), regulating adaptive immune responses and participating in other relevant pathways [8, 9]. Moreover, in various models and experiments, NK cells can distinguish between abnormal and healthy cells, exhibiting more specific anti-tumor cytotoxicity, thereby reducing off-target complications [10, 11]. Currently, numerous studies and drugs target NK cell-related pathways to inhibit tumors [12, 13]. Ineffective activation of NK cells is one of the reasons hindering the long-term development of adaptive immunity against lung cancer. Strategies to reactivate NK cells, possibly beneficial for the clinical outcomes of lung cancer patients, deserve further investigation, especially in combination with other therapies [14]. Therefore, exploring the potential mechanisms related to immune escape in lung cancer NK cells warrants in-depth research.

Long non-coding RNAs (lncRNAs) are a class of non-coding RNAs with lengths exceeding 200 nucleotides, playing diverse roles in the regulation of gene expression. An increasing body of research suggests that dysregulated lncRNA expression is associated with the occurrence and progression of human tumors [15]. LINC00665, a novel lncRNA, exhibits abnormal expression in various human cancers, including lung cancer [16]. Elevated expression of LINC00665 has been shown in lung cancer tissues and is associated with TNM staging, lymph node metastasis, and tumor size. Its high expression promotes in vitro proliferation, migration, invasion of lung cancer cells, and regulates cell cycle arrest and apoptosis [17, 18]. However, the biological role of LINC00665 in immune escape in lung cancer remains unclear, and there are currently no reported studies on this aspect. In this study, we explore the impact and mechanisms of LINC00665 on lung cancer development and NK cell cytotoxicity, aiming to provide reference for finding drugs that can be used to inhibit immune escape and tumor development.

## Materials and methods

### Clinical sample

This study included 84 lung cancer patients who underwent surgical treatment at our hospital from June 2020 to June 2022 as research subjects. Based on the occurrence of metastasis, these 84 lung cancer patients were divided into the non-metastasis group (*n* = 58) and the metastasis group (*n* = 26). There were no significant differences in clinical baseline data, including gender, age, BMI, etc., between the two groups. Tumor tissues and matched normal lung tissues were collected from all patients and analyzed using qRT-PCR. All tumor and matched normal tissues were confirmed by histopathological examination. This study was approved by our hospital’s ethics committee and adheres to the principles of the Helsinki Declaration. Informed consent forms were signed by all participants before their involvement. The baseline of the LC patients is displayed in Supplementary Table 1.

### RNA extraction and quantitative reverse transcription polymerase chain reaction (qRT-PCR)

RNA isolation from tissues or cells was performed using Trizol (Invitrogen, Carlsbad, CA, USA). The purity and concentration of the extracted RNAs were assessed using a NanoDrop 2000 spectrophotometer (Thermo Fisher Scientific, Waltham, USA). A total of 500 ng of RNA was used to synthesize cDNA with the PrimeScript reverse transcriptase reagent kit (Takara, Shiga, Japan). Subsequently, quantitative real-time polymerase chain reaction (qRT-PCR) was carried out on the Light 7500 Real-Time PCR System (Applied Biosystems, USA) using SYBR Premix Ex TaqII (Takara) to determine the indicated RNA levels. The thermal cycling conditions were set as follows: 95 °C for 5 min, followed by 40 cycles of 95 °C for 10 s, 58 °C for 30 s, and 72 °C for 1 min. Primer sequences are provided in Table1. GAPDH served as a normalized reference. The relative RNA levels were analyzed using the 2-ΔΔCt method. All primers were designed and synthesized by Sangon (Shanghai, China). Primers were exhibited in Table 1.

**Table 1:** qRT-PCR primer sequences

Gene	Forward 5’-3’	Reverse 5’-3’
LINC00665	GTTTCCTGACCTCTGACCCG	CCCACATGGTAGTCGATCCG
TGF-β	TACCTGAACCCGTGTTGCTCTC	GTTGCTGAGGTATCGCCAGGAA
IL-10	TCTCCGAGATGCCTTCAGCAGA	TCAGACAAGGCTTGGCAACCCA
IL-1β	CCACAGACCTTCCAGGAGAATG	GTGCAGTTCAGTGATCGTACAGG
IFN-γ	GAGTGTGGAGACCATCAAGGAAG	TGCTTTGCGTTGGACATTCAAGTC
IL-2	AGAACTCAAACCTCTGGAGGAAG	GCTGTCTCATCAGCATATTCACAC
TNF-α	CTCTTCTGCCTGCTGCACTTTG	ATGGGCTACAGGCTTGTCACTC
GAPDH	CACCCACTCCTCCACCTTTGA	ACCACCCTGTTGCTGTAGCCA

### Cell culture

The human lung cancer cell lines A549, H358, H1299, Calu-3, and normal human lung epithelial cells BEAS-2B (American Type Culture Collection, Manassas, VA, USA) were cultured in DMEM/RPMI-1640/Ham’s F-12 K medium containing 10% FBS (GIBCO) and 1% penicillin-streptomycin at 37 °C in a 5% CO2 humidified incubator. The human NK cell line NK92 cells (ATCC) were cultured in MEMa supplemented with 12.5% FBS, 2 mM L-glutamine, and 12.5% horse serum (Gibco). For NK92 cell activation, cells were stimulated with 100 U/mL IL-2 (Gibco, PHC0023) for 24 h. For co-culture experiments, the activated NK-92 cells were co-cultured with A549 or H1299 cells at a ratio of 10:1 for 4 h.

### Cell transfection

The lentiviral vectors pSIH1-H1-copGFP (8,619,936, BioVector Science Lab Inc., Beijing, China) and pLV-EGFP-N (VL3211, Inovogen Tech Co., Ltd., Beijing, China) were utilized to construct lentiviral short hairpin RNAs (shRNAs) targeting LINC00665 (sh-LINC00665) and lentiviral vectors for overexpressing HHLA2 (oe-HHLA2). These constructs were separately used to infect A549/H1299 lung cancer cells of the corresponding groups. Transfections were carried out using Lipofectamine 2000 (Invitrogen, Carlsbad, CA, USA) according to the manufacturer’s instructions. The sequences of the shRNAs used in this study are provided in Table 2.

**Table 2:** Sequences of shRNAs used in this study

Definition	sequences
sh-LINC00665-#1	5’-GCAGGTGGCCTCCAGGTGCAAAGTTCAAGAGACTTTGCACCTGGAGGCCACCTGCTTTTTT-3’
sh-LINC00665-#2	5’-GGGACGCTGGAGGGTCAGCAGTTCAAGAGACTGCTGACCCTCCAGCGTCCCTTTTTT-3’
sh-LINC00665-#3	5’-GGCCACGTGCCTGCCGGACATTTCAAGAGAATGTCCGGCAGGCACGTGGCCCTTTTTT-3’
sh-NC	5’-UUCUCCGAACGUGUCACGUTT-3’

### CCK-8

Cells were seeded at a density of 5000 cells per well in 96-well plates (Solarbio, Beijing, China). Subsequent to transfection based on experimental requirements, cells were cultured and subjected to detection using the Cell Counting Kit-8 assay every 24 h. The optical density (OD450) values were measured and analyzed using a microplate reader (Bio-RAD, USA).

### Flow Cytometric Analysis (FACS)

After 24 h of transfection, A549/H1299 cells from each treatment group were collected, and staining for apoptosis was conducted using the Annexin V-FITC Apoptosis Detection Kit (BD Bioscience, Oxford, UK) following the manufacturer’s guidelines. Cell apoptosis rates were assessed using flow cytometry. In addition, following a protocol referenced from previous research [19], NK92 cells were co-cultured with A549/H1299 cells at a 1:1 ratio for 5–6 days. Flow cytometry was employed to evaluate NK92 cell proliferation (Cell-Trace or Ki67), degranulation (CD107a), and IFN-γ production. In brief, cell surface and intracellular protein staining were performed using antibodies against IFNγ, Ki67 (proliferation), and CD107a (degranulation). The assessment of NK cell phenotype and function was conducted following fixation and permeabilization with the eBioscience kit according to the manufacturer’s instructions. NK cells were labeled with CellTrace proliferation dye (ThermoFisher Scientific) before co-culture with A549/H1299. Fixable live/dead cell dye (ThermoFisher Scientific) was used to determine viable cells. All cells were acquired using the LSRFortessa flow cytometer and analyzed with FlowJo 10.7.

### Transwell assays

In the transwell experiment with A549/H1299 cells, we utilized transwell chambers (Corning, USA) coated with Matrigel. A total of 1 × 10^4 cells were initially cultured in the upper transwell chamber with only DMEM, while the lower chamber was filled with 10% FBS (fetal bovine serum) and DMEM. After 24 h, the medium and noninvasive cells were removed from the upper chamber, and the invasive cells were subsequently counted under an optical microscope (Olympus, Japan). Additionally, NK cell migration was assessed in transwell assays designed to permit only active migration of NK cells, which were placed on the upper insert, towards the bottom where A549/H1299 cells were cultured. The number of NK cells was determined by relative CD56 percentages using flow cytometry.

### Cytotoxicity assay

After stimulating NK92 cells with 100 U/mL IL-2 (Gibco, PHC0023) for 24 h, activated NK-92 cells were co-cultured with A549/H1299 cells at a ratio of 10:1 for 4 h. The CytoTox96 Non-Radioactive Cytotoxicity Assay Kit (Promega, Madison, WI, USA) was then used to measure the lactate dehydrogenase (LDH) levels in A549/H1299 cells. Briefly, cells were plated at a density of 2.3 × 10^5 cells/mL in a 96-well plate. Fifty microliters of CytoTox96 Reagent was added to each well and incubated for 30 min at room temperature. This was followed by the addition of Stop Solution (50 µL). The absorbance at 490 nm (A490) was determined using a microplate reader (Bio-Rad). Percent cytotoxicity was calculated as follows: Specific lysis (%) = [optical density (OD) experimental group – OD target cell natural release control] / (OD target cell maximum release control – OD target cell natural release control) × 100. The maximum release was measured after treatment with the lysis agent provided by the manufacturer.

### ELISA

The levels of IFN-gamma and TNF-alpha in the cell culture medium were measured using ELISA kits (BMS228 and BMS223-4, Invitrogen). In brief, the culture medium was collected and centrifuged at 1400 rpm for 1 min. The ELISA assay was conducted following the manufacturer’s instructions, and the absorbance at 450 nm (A450) was determined using a microplate reader (Bio-Rad).

### RNA-FISH subcellular localization

RNA-FISH was employed to investigate the cellular localization of LINC00665. DNA oligo probes for LINC00665 (labeled with FAM) were obtained from GenePharma (Shanghai, China). A549 cells (1 × 10^5) were seeded in a 24-well plate. After 24 h, the culture medium was removed, and cells were washed with PBS three times. Cells were fixed using paraformaldehyde and prehybridized with PBS containing 0.5% Triton X-100. Subsequently, the cells were hybridized with LINC00665 probes in hybridization buffer overnight at 42 °C. DAPI (Beyotime) was used for nuclear counterstaining. Finally, observation and imaging were performed under a Leica SP5 confocal microscope (Leica Microsystems, Mannheim, Germany).

### Nucleus-cytoplasm separation experiment

According to the manufacturer’s instructions, the NE-PER Nuclear and Cytoplasmic Extraction Reagents kit (Thermo Scientific) was used to extract nuclear and cytoplasmic fractions separately. The expression of LINC00665 in the nuclear and cytoplasmic extracts was then detected by RT-qPCR.

### RNA immunoprecipitation (RIP)

Anti-TCF7 antibody (PAB27159, Wuhan AmyJet Scientific Inc.) was used for RIP analysis of E2F1, with IgG as a control. RIP experiments were conducted using the Magna RIP™ RNA-binding protein immunoprecipitation kit (Millipore, Merck KGaA, Germany) following the manufacturer’s guidelines. The purified RNA was then subjected to qRT-PCR to detect co-precipitated LINC00665 and HHLA2 mRNA.

### RNA fish and immunefluorescence

Briefly, H1299 or A549 cells incubated with anti-α-TCF7 or HHLA2 antibody (1:200, CST) and then incubated with Alexa Fluor 555-conjugated secondary antibody (1:10000, A32727, Thermo Fisher). The sections were dehydrated with ethanol and rehydrated in 50% formamide and then hybridized with a 20 nM 5′-digoxigenin-labeled LINC00665 probe (Ribobio) and anti-digoxigenin-FITC. DAPI was used for counterstaining the nuclei, and images were observed with laser scanning confocal microscopy (LSM710, Zeiss).

### Western blot

Protein was extracted using radioimmunoprecipitation assay buffer (Thermo Fisher Scientific), and the concentration was determined with a bicinchoninic acid kit (Beyotime). Thirty micrograms (30 µg) of protein were separated by 10% SDS-PAGE, then transferred to a PVDF membrane (Millipore, Bedford, MA, USA). After blocking in 5% skim milk for 2 h, the membrane was incubated overnight at 4 °C with primary antibodies against TCF7 (1:1000, ab159646), HHLA2 (1:500, ab214327), and β-actin (1:5000, ab6276) (all antibodies were obtained from Abcam). Following TBST washing, an HRP-conjugated secondary antibody (ab205718, 1:2000) was added and incubated at room temperature for 1 h, followed by visualization using ECL reagent (EMD Millipore, USA). Image J software (Media Cybernetics, USA) was used for grayscale quantification of bands in the western blot images, with β-actin serving as an internal reference. Each experiment was repeated three times. Original uncropped blot images was displayed in Supplementary File 1.

### Animal study

Female NOD-SCID mice (4 to 6 weeks old, n = 6 per group) were purchased from Beijing Weitong Lihua Experimental Animal Technology Co., Ltd. (Beijing, China). The transfected A549 cells were subcutaneously injected into the flank of the mice. Tumor size was monitored every 7 days and calculated using the formula: Volume = 1/2 × length × width^2. IL-2 activated NK92 cells were injected through the tail vein on day 3 and 7 post-inoculation. On day 28, the xenograft tumors were harvested and weighed. All animal experiments were conducted in accordance with established protocols and approved by the Animal Protection and Use Committee of our institution, adhering to the “Guidelines for the Care and Use of Laboratory Animals.

### Immunohistochemistry (IHC)

The deparaffinized sections underwent antigen retrieval. After blocking with 1% BSA, the slides were incubated with the Ki67 antibody (ab15580), followed by incubation with an HRP-conjugated secondary antibody. The signal was detected using the DAB color development kit (Beyotime).

### Statistical analysis

Data statistical analysis and plotting were conducted using SPSS 21.0 (SPSS, Inc., Chicago, IL, USA) and GraphPad Prism 8.01 software (GraphPad Software Inc., San Diego, CA, USA). Measurement data with a normal distribution, confirmed by the Shapiro-Wilk test, are presented as mean ± standard deviation. Pairwise/independent sample t-tests were used for comparisons between two groups. One-way ANOVA analysis was employed for comparisons among multiple groups, followed by Tukey’s multiple comparisons test for post hoc analysis. A significance level of *P* < 0.05 was considered statistically significant.

## Results

### High expression of LINC00665 in patients with metastatic Lung Cancer and its correlation with Immune escape-related cytokine levels

**Fig. 1:**
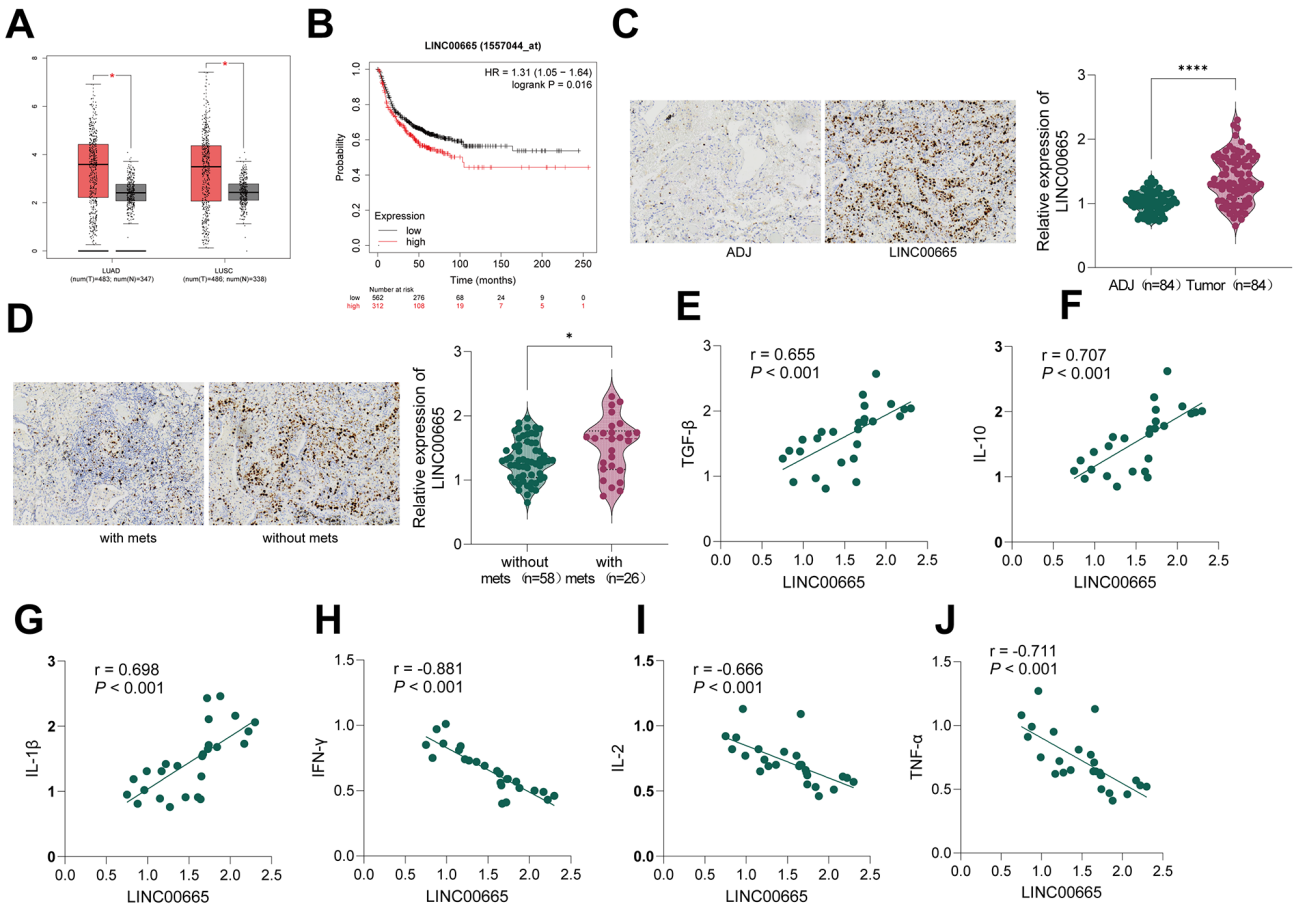
LINC00665 is Highly Expressed in Patients with Metastatic Lung Cancer and Correlates with Levels of Immune Escape-Related Cytokines. (**A**) TCGA database predictions for the expression of LINC00665 in LUAD/LUSC and (**B**) the relationship between LINC00665 and the prognosis of LUAD/LUSC. (**C**) qRT-PCR detection of LINC00665 expression in tumor tissues and adjacent tissues of lung cancer patients. (**D**) qRT-PCR detection of LINC00665 expression in tumor tissues of patients with metastatic and non-metastatic lung cancer. Pearson analysis of the correlation between LINC00665 in metastatic lung cancer and immune inhibitory cytokines (**E**) TGF-β, (**F**) IL-10, (**G**) IL-1β, as well as anti-tumor cell cytokines (**H**) IFN-γ, (**I**) IL-2, and (**J**) TNF-α. Data are presented as mean ± standard deviation. Comparison between the two groups in (C) was performed using paired t-tests, and comparison between the two groups in (D) was performed using independent sample t-tests. *** indicates *P* < 0.001

Analysis of The Cancer Genome Atlas (TCGA) database (https://ualcan.path.uab.edu/analysis.html) revealed a significant upregulation of LINC00665 in both lung adenocarcinoma (LUAD) and lung squamous cell carcinoma (LUSC) (both *P* < 0.05, Fig. 1A). Moreover, its high expression was associated with poor prognosis in patients (both *P* < 0.05, Fig. 1B). To investigate whether LINC00665 is associated with lung cancer metastasis and immune escape, we collected tumor tissues and corresponding adjacent tissues from 84 lung cancer patients. Based on the occurrence of metastasis, patients were divided into the non-metastatic group (*n* = 58) and metastatic group (*n* = 26). qRT-PCR results demonstrated a significant increase in LINC00665 expression in tumor tissues compared to adjacent tissues (both *P* < 0.05, Fig. 1C), and metastatic lung cancer patients exhibited significantly higher levels of LINC00665 compared to non-metastatic lung cancer patients (both *P* < 0.05, Fig. 1D).

Furthermore, we analyzed the correlation between LINC00665 levels in metastatic lung cancer patients and immune-suppressive cytokines (TGF-β, IL-10, and IL-1β) as well as anti-tumor cytokines (IFN-γ, IL-2, and TNF-α). Pearson analysis revealed a significant positive correlation between LINC00665 in metastatic lung cancer and the immune-suppressive cytokines TGF-β, IL-10, and IL-1β (all *P* < 0.05, Fig. 1E-G), while a significant negative correlation was observed with the anti-tumor cytokines IFN-γ, IL-2, and TNF-α (all *P* < 0.05, Fig. 1H-J). These results suggest that the high expression of LINC00665 is associated with metastatic lung cancer and correlates with cytokine levels related to immune escape, indicating its potential involvement in the regulation of lung cancer metastasis and immune escape processes.

### Knockdown of LINC00665 suppresses proliferation, Migration, and Invasion while promoting apoptosis in Lung Cancer cells

**Fig. 2:**
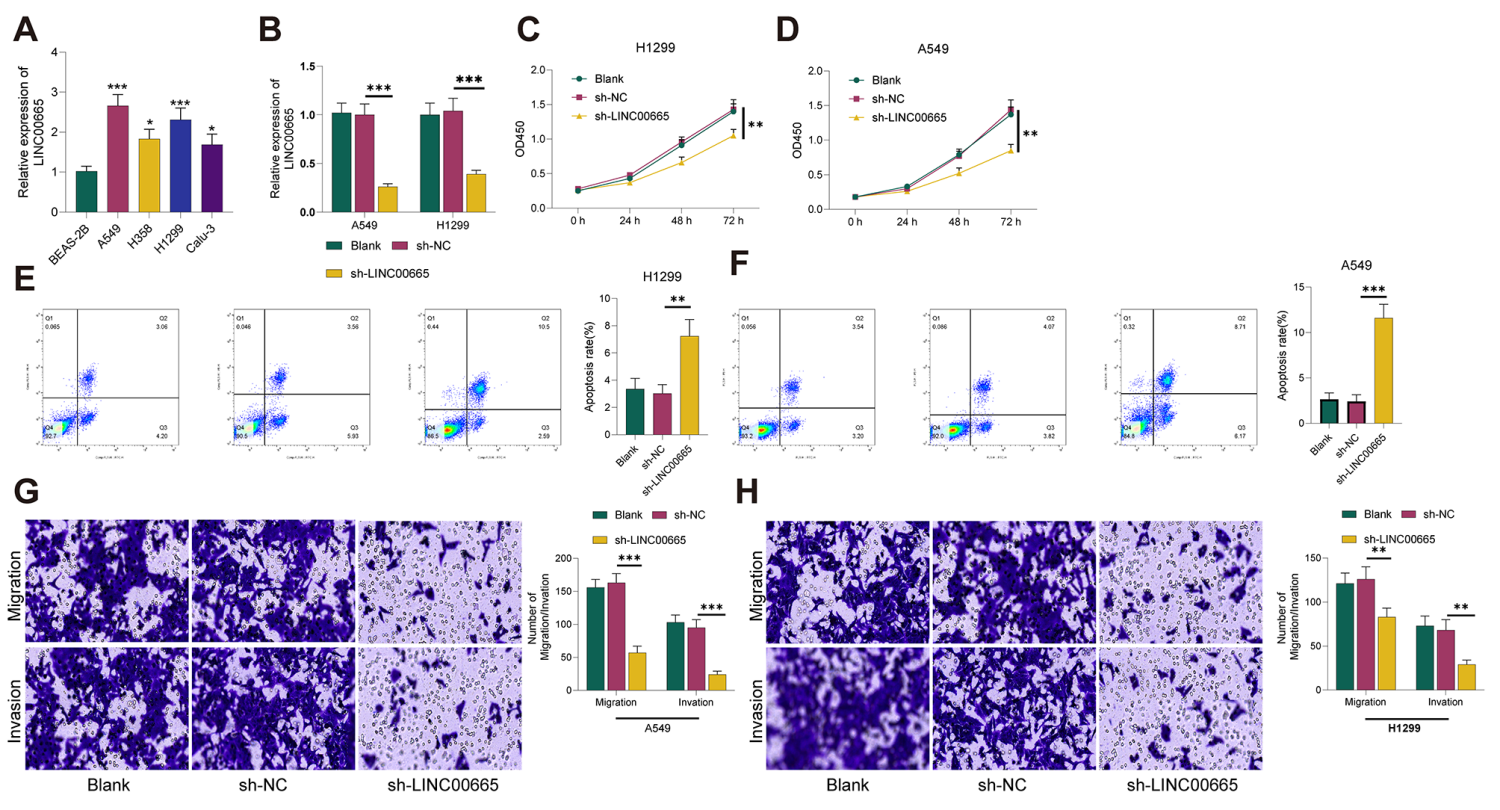
Knockdown of LINC00665 Inhibits Proliferation, Migration, and Invasion of Lung Cancer Cells and Promotes Apoptosis. (**A**) qRT-PCR detection of LINC00665 expression in different lung cancer cell lines. Lentivirus transfection of sh-LINC00665 into lung cancer cells, followed by (**B**) qRT-PCR to detect changes in LINC00665 expression. (**C/D**) CCK-8 assay to measure cell viability. (**E/F**) Flow cytometry to detect cell apoptosis. (**G/H**) Transwell assay to measure invasion and migration. The cell experiments were repeated three times, and data are presented as mean ± standard deviation. Multiple group comparisons were performed using one-way ANOVA, and post hoc analysis was conducted using Tukey’s multiple comparisons test. ** indicates *P* < 0.01, *** indicates *P* < 0.001

Furthermore, we conducted in vitro cell experiments to investigate the impact and underlying mechanisms of LINC00665 on lung cancer metastasis and immune escape. Consistent with clinical results, qRT-PCR analysis revealed that LINC00665 was significantly upregulated in lung cancer cell lines (A549, H358, H1299, Calu-3) compared to normal human lung epithelial cells (BEAS-2B) (all *P* < 0.05, Fig. 2A). A549 and H1299, which exhibited the highest relative expression, were chosen for subsequent experiments. We employed lentiviral transfection of sh-LINC00665 to knock down its expression in lung cancer cells. qRT-PCR results demonstrated a significant reduction in LINC00665 expression in lung cancer cells in the sh-LINC00665 group compared to the sh-NC group (all *P* < 0.001, Fig. 2B), indicating successful cell transfection. Subsequently, we assessed the impact of LINC00665 knockdown on the malignant behavior of lung cancer cells using CCK-8, flow cytometry, and Transwell assays. The results showed that LINC00665 knockdown significantly inhibited the proliferation of lung cancer cells compared to the sh-NC group (all *P* < 0.01, Fig. 2C-D). Additionally, it promoted cell apoptosis (all *P* < 0.01, Fig. 2E-F) and enhanced migration and invasion capabilities (all *P* < 0.001, Fig. 2G-H). These findings indicate that the knockdown of LINC00665 suppresses the malignant behavior of lung cancer cells.

### Knockdown of LINC00665 enhances NK cell cytotoxicity against Lung Cancer cells

**Fig. 3:**
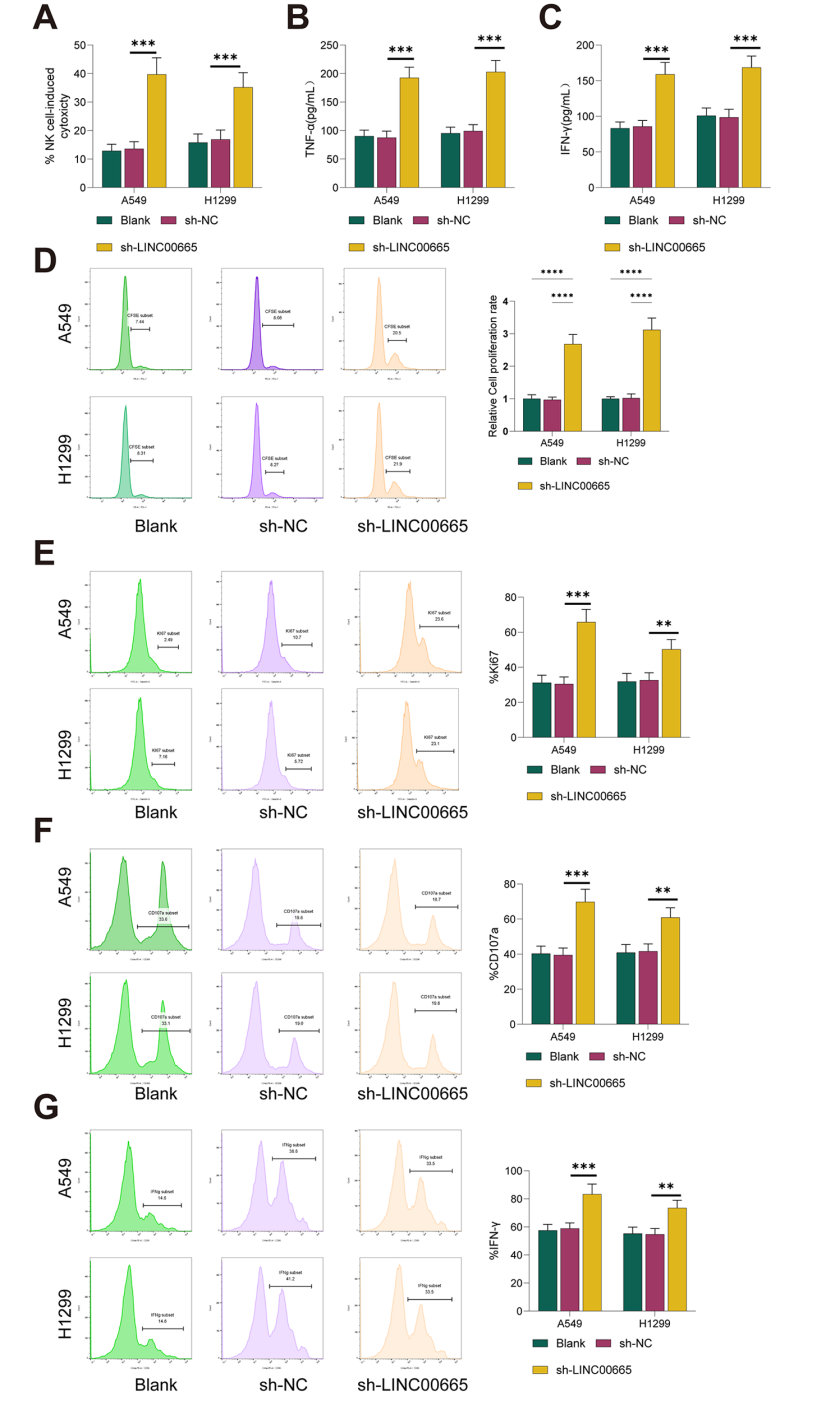
Knockdown of LNC00665 Promotes NK Cell Cytotoxicity Against Lung Cancer Cells. NK92 cells were stimulated with IL-2, and then activated NK92 cells (effector cells) were co-cultured with A549/H1299 cells in each treatment group. (**A**) Detection of lactate dehydrogenase (LDH) levels in A549/H1299 cells using the CytoTox96 non-radioactive cytotoxicity assay (Promega, Madison, WI, USA) to assess NK cell cytotoxicity against lung cancer cells. ELISA was used to measure the secretion of (**B**) IFN-γ and (**C**) TNF-α in the cell culture supernatant of the co-culture system. (**D**) NK cell migration was evaluated in transwell assays allowing for only active migration of NK cells, laid onto the upper insert, towards the bottom where A549/H1299 cells were cultured. (**E-G**) Purified NK cells were co-cultured with A549/H1299 cells for 5–6 days at a 1:1 ratio and evaluated for proliferation (Cell-Trace or Ki67), IFN-γ production, and degranulation (CD107a). NK cell IFN-γ and degranulation were assessed following stimulation with PMA and ionomycin for 6 h prior to staining. The cell experiments were repeated three times, and data are presented as mean ± standard deviation. Multiple group comparisons were performed using one-way ANOVA, and post hoc analysis was conducted using Tukey’s multiple comparisons test. ** indicates *P* < 0.01, *** indicates *P* < 0.001

Next, we investigated the impact of LINC00665 on NK cell cytotoxicity. NK92 cells were stimulated with IL-2, and then the activated NK92 cells (effector cells) were co-cultured with A549/H1299 cells (target cells) in various treatment groups. The cytotoxicity of NK cells against lung cancer cells was assessed by measuring lactate dehydrogenase (LDH) released from lysed target cells. As shown in Fig. 3A, silencing LINC00665 in A549/H1299 cells significantly enhanced NK92 cell cytotoxicity (all *P* < 0.001). Simultaneously, we measured the secretion of IFN-γ and TNF-α in the co-culture supernatant using ELISA. The results in Fig. 3B-C demonstrated that silencing LINC00665 in A549/H1299 cells significantly increased the release of IFN-γ and TNF-α (all *P* < 0.001). Meanwhile, in the in vitro transwell migration assay, the knockdown of LINC00665 significantly inhibited the migration of NK cells (all *P* < 0.001, Fig. 3D), indicating that LINC00665-silenced lung cancer cells may inhibit the infiltration of NK cells into lung cancer tumors. Additionally, we co-cultured NK92 cells labeled with proliferation dye CellTrace with A549/H1299 cells and assessed the proliferation (Cell-Trace or Ki67), degranulation (CD107a), and IFN-γ production of NK92 cells by flow cytometry. The results showed that knocking down LINC00665 enhanced the proliferation (all *P* < 0.01, Fig. 3E), degranulation (all *P* < 0.01, Fig. 3F), and IFN-γ production (all *P* < 0.01, Fig. 3G) of NK cells. These findings suggest that the knockdown of LINC00665 promotes NK cell cytotoxicity against lung cancer cells.

### LINC00665 upregulates HHLA2 expression by recruiting transcription factor TCF7

**Fig. 4:**
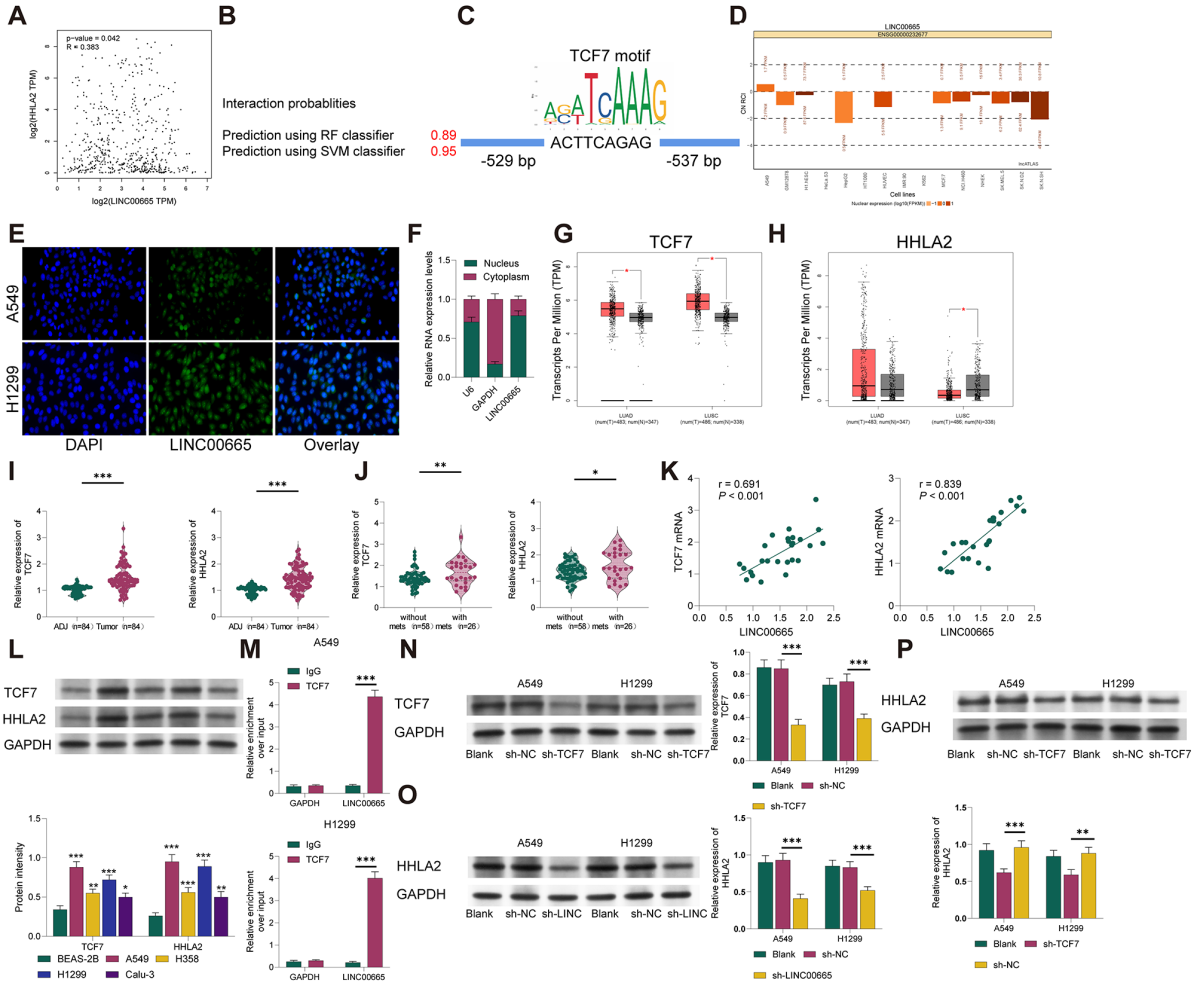
LINC00665 Upregulates HHLA2 Expression in Lung Cancer Cells by Recruiting Transcription Factor TCF7. (**A**) Analysis of the correlation between LINC00665 and downstream genes using the R Corr package in the GSE138682 gene expression microarray dataset. (**B**) Analysis of LINC00665 targeting binding sites with the transcription factor TCF7 using the catRAPID website. (**C**) Prediction of TCF7 binding sites on the HHLA2 promoter using the JASPAR website. (**D**) Prediction of the main nuclear localization of LINC00665 using the LncATLAS database. (**E**) RNA-FISH and (**F**) nuclear-cytoplasmic fractionation experiments confirming the predominant nuclear localization of NEAT1 in A549 cells. (**G**) TCGA database analysis showing significant upregulation of TCF7 in LUAD. (**H**) Analysis from the ENCORI Pan-Cancer Analysis Platform database indicating significant upregulation of HHLA2 in LUSC. (**I**) qRT-PCR detection of LINC00665 expression in tumor and adjacent tissues of lung cancer patients. (**J**) qRT-PCR detection of LINC00665 expression in tumor tissues of patients with and without metastasis. (**K**) Pearson analysis of the correlation between LINC00665 and TCF7 and HHLA2 mRNA levels in metastatic lung cancer. (**L**) Western blot analysis of TCF7 and HHLA2 expression in different lung cancer cell lines. (**M**) RIP experiment validating the binding ability of LINC00665 with TCF7. (**N**) Western blot detection of (N) TCF7 and (**O/P**) HHLA2 expression. Cell experiments were repeated three times, and data are presented as mean ± standard deviation. Multiple group comparisons were performed using one-way ANOVA, and post hoc analysis was conducted using Tukey’s multiple comparisons test. * indicates *P* < 0.05, ** indicates *P* < 0.01, *** indicates *P* < 0.001

Furthermore, we explored the downstream regulatory mechanism of LINC00665 in the modulation of malignant behaviors in lung cancer cells and the cytotoxicity of NK cells. Through analysis of the gene expression microarray GSE138682 using the R Corr package, we found that LINC00665 had the highest correlation with HHLA2 (Fig. 4A). HHLA2 has been reported to inhibit NK cell cytotoxicity [20]. Additionally, analysis using the catRAPID website revealed that LINC00665 has binding sites targeting the transcription factor TCF7 (Fig. 4B). JASPAR website analysis indicated that TCF7 has binding sites on the promoter of HHLA2 (Fig. 4C). LncATLAS database predictions suggested that LINC00665 primarily localizes to the cell nucleus (Fig. 4D), and RNA-FISH and nuclear-cytoplasmic separation experiments confirmed that LINC00665 is mainly located in the nucleus of A549 cells (Fig. 4E-F). TCGA database analysis showed significant upregulation of TCF7 in LUAD and HHLA2 in LUSC (*P* < 0.05, Fig. 4G-H). Based on this, we hypothesized that LINC00665 could promote the transcription of HHLA2 in lung cancer cells by recruiting the transcription factor TCF7, thereby participating in the regulation of NK cell cytotoxicity.

To validate this hypothesis, we first used qRT-PCR to detect the expression of TCF7 and HHLA2 in tumor tissues of lung cancer patients and metastatic lung cancer patients. The results showed that, compared to adjacent tissues, the expression of TCF7 and HHLA2 mRNA in tumor tissues of lung cancer patients was significantly elevated (all *P* < 0.001, Fig. 4I). Furthermore, compared to non-metastatic lung cancer, TCF7 and HHLA2 mRNA levels were significantly increased in metastatic lung cancer patients (all *P* < 0.05, Fig. 4J). In addition, TCF7 and HHLA2 mRNA levels in metastatic lung cancer patients were significantly positively correlated with LINC00665 levels (all *P* < 0.001, Fig. 4K). Western blot analysis showed that TCF7 and HHLA2 were significantly upregulated in different lung cancer cell lines (all *P* < 0.05, Fig. 4L). Subsequently, we verified the binding ability of LINC00665 to TCF7 through RIP experiments. The results showed that compared to the IgG immunoprecipitation group, the enrichment of LINC00665 in the TCF7 immunoprecipitation group significantly increased (all *P* < 0.001, Fig. 4M), suggesting a binding relationship between LINC00665 and TCF7. Furthermore, we validated the roles of LINC00665 and TCF7 in regulating HHLA2 expression. Western blot results demonstrated that knocking down LINC00665 or TCF7 (all *P* < 0.001, Fig. 4N) significantly decreased the expression of HHLA2 in lung cancer cells (all *P* < 0.05, Fig. 4O-P). These results indicate that LINC00665 can upregulate the expression of HHLA2 in lung cancer cells by recruiting the transcription factor TCF7. To further validate the binding relationship between LINC00665 and TCF7, we conducted fluorescence co-localization experiments using a 5’-digoxigenin probe and anti-TCF7. We observed significant co-localization of LINC00665 and TCF7 in H1299 or A549 cells. However, we did not observe significant binding between LINC00665 or TCF7 and HHLA2. This is likely because LINC0065 or TCF7 primarily localize in the cell nucleus, while HHLA2 localizes on the cell membrane (Fig S1A ~ C).

### Overexpression of HHLA2 partially reverses the Inhibitory Effect of LNC00665 Knockdown on the malignant behavior of Lung Cancer cells

**Fig. 5:**
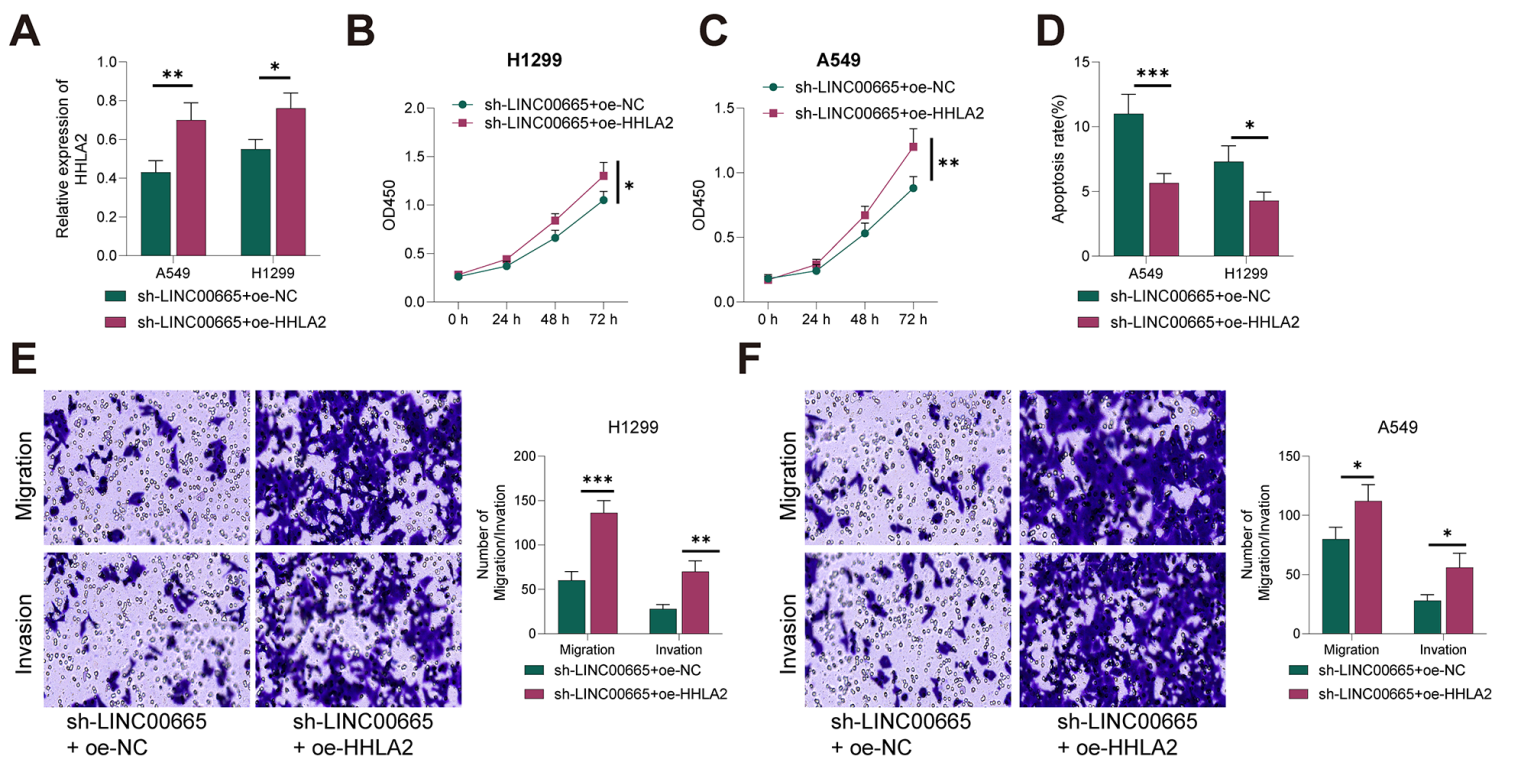
Partial Reversal of the Inhibitory Effect of LINC00665 Knockdown on the Malignant Behavior of Lung Cancer Cells by Overexpressing HHLA2. (**A**) Western blot analysis of HHLA2 expression. (**B/C**) CCK-8 assay measuring cell viability. (**D**) Flow cytometry analysis of cell apoptosis. (**E/F**) Transwell assay evaluating invasion and migration. Cell experiments were repeated three times, and data are presented as mean ± standard deviation. The comparison between the two groups was performed using an independent sample t-test. * indicates *P* < 0.05, ** indicates *P* < 0.01, *** indicates *P* < 0.001

Earlier, we confirmed that LINC00665 can upregulate the expression of HHLA2 in lung cancer cells by recruiting the transcription factor TCF7. We further investigated whether LINC00665 could regulate the malignant behavior of lung cancer cells by upregulating HHLA2. We performed cell transfection to simultaneously knock down LINC00665 and overexpress HHLA2 in lung cancer cells (both *P* < 0.05, Fig. 5A) and examined their impact on the malignant behavior of lung cancer cells. The results showed that overexpression of HHLA2 partially reversed the inhibitory effects of LINC00665 knockdown on the proliferation, migration, invasion, and promotion of apoptosis in lung cancer cells (all *P* < 0.05, Fig. 5B-F).

### Overexpression of HHLA2 partially reverses the enhanced cytotoxic activity of NK cells Induced by LINC00665 Knockdown

**Fig. 6:**
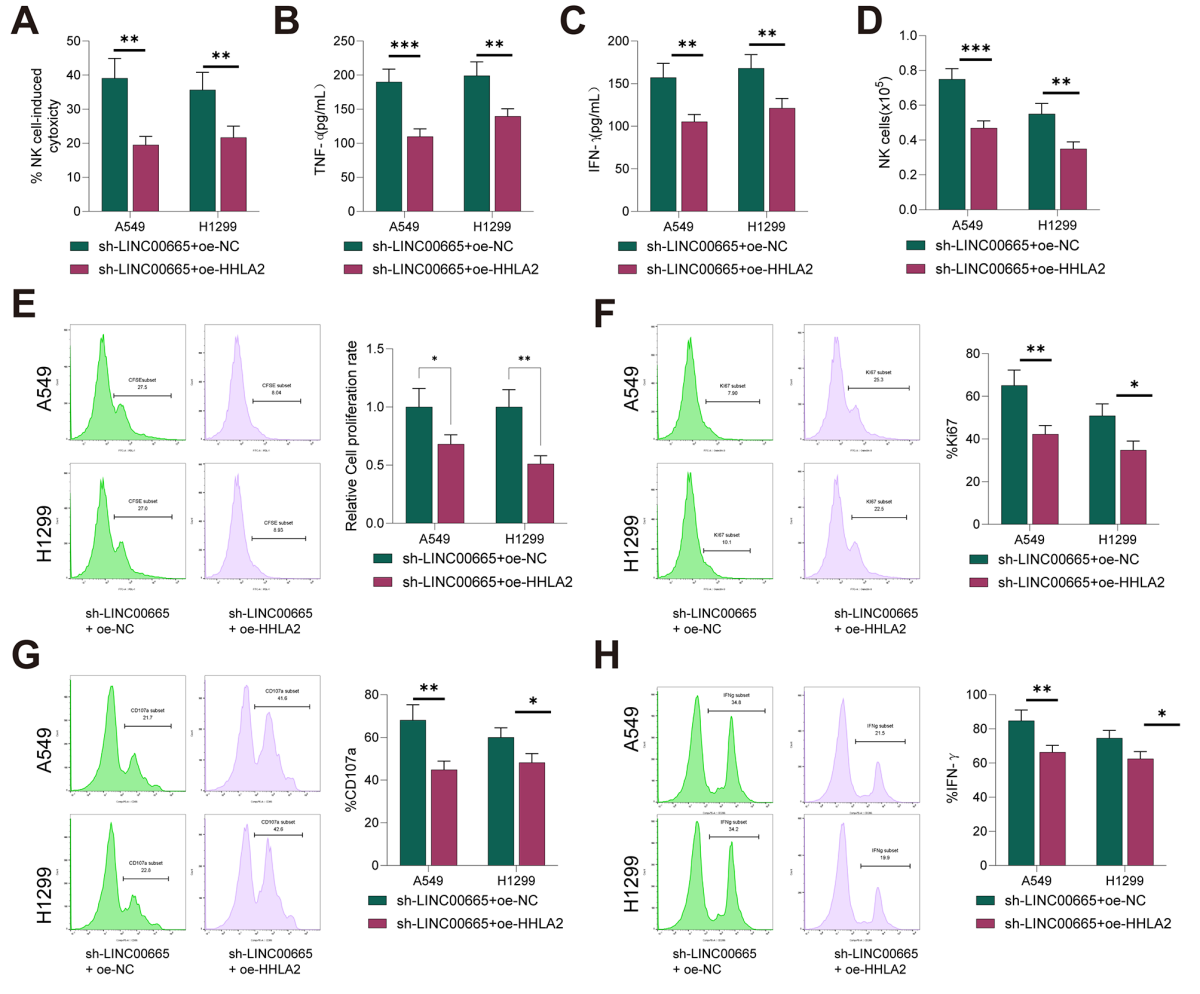
Partial Reversal of the Enhanced NK Cell Cytotoxicity Against Lung Cancer Cells by LINC00665 Knockdown Through Overexpression of HHLA2. (**A**) Western blot analysis of HHLA2 expression. (**B**) CCK-8 assay measuring cell viability. (**C**) Flow cytometry analysis of cell apoptosis. (**D**) Transwell assay evaluating invasion and migration. (**E-H**) Proliferation (Cell-Trace or Ki67), degranulation (CD107a), and IFN-γ production of NK92 cells co-cultured with A549/H1299 cells. Cell experiments were repeated three times, and data are presented as mean ± standard deviation. The comparison between the two groups was performed using an independent sample t-test. * indicates *P* < 0.05, ** indicates *P* < 0.01, *** indicates *P* < 0.001

We also investigated whether LINC00665 could regulate the cytotoxic activity of NK cells by upregulating HHLA2. The results showed that after overexpressing HHLA2, the enhanced cytotoxicity of NK92 cells and the increased release of IFN-γ and TNF-α induced by LINC00665 knockdown were partially reversed (all *P* < 0.05, Fig. 6A-C). Moreover, compared to the sh-LINC00665 + oe-NC group, overexpression of HHLA2 inhibited the proliferation, migration, degranulation, and IFN-γ production of NK cells (all *P* < 0.05, Fig. 6D-H). These results indicate that overexpression of HHLA2 partially reverses the enhanced cytotoxic activity of NK cells induced by LINC00665 knockdown.

### In vivo, depletion of LINC00665 suppresses Tumor Growth and enhances sensitivity of NK cells

**Fig. 7:**
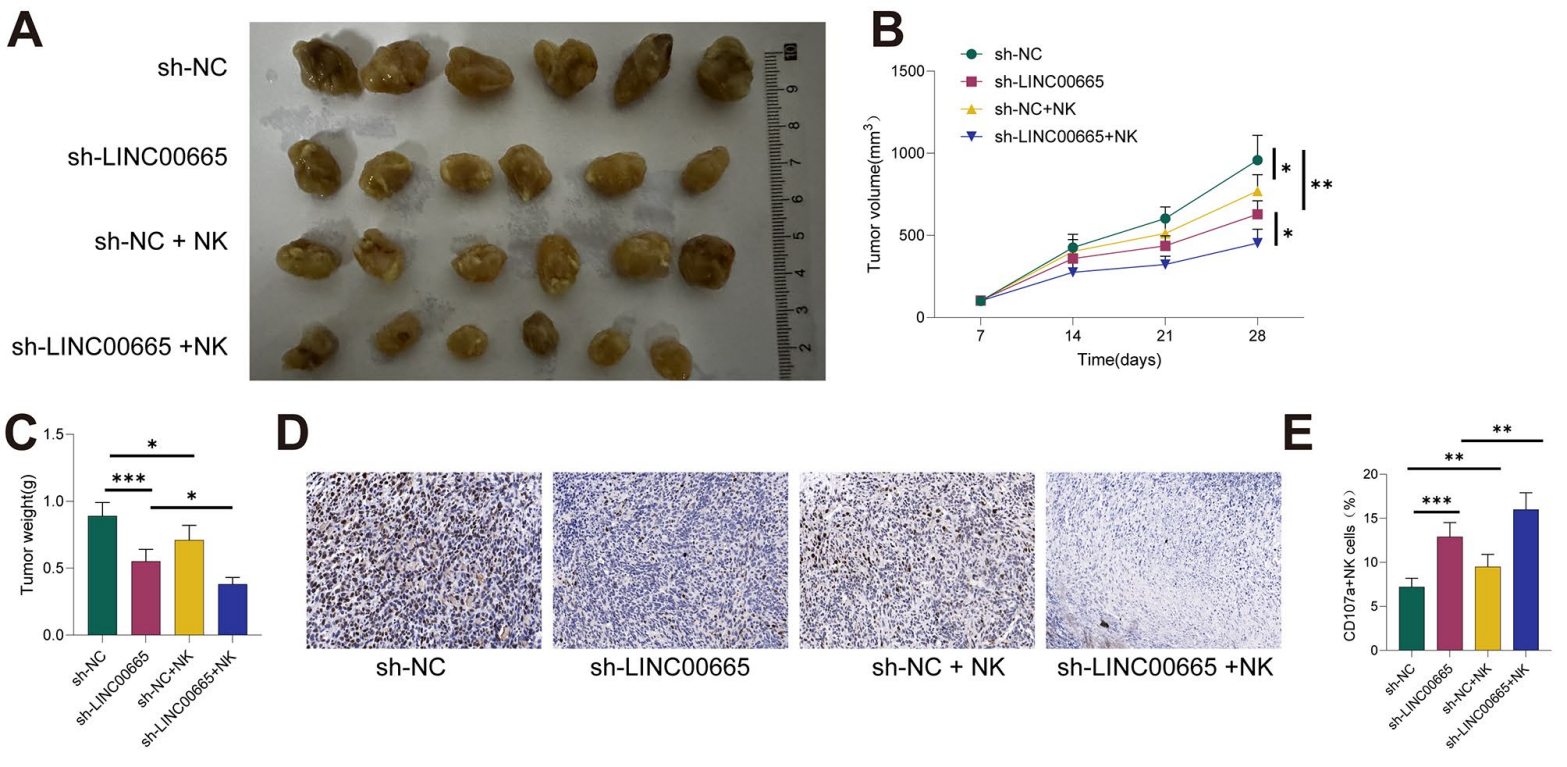
In Vivo Deletion of LINC00665 Suppresses Tumor Growth and Enhances NK Cell Sensitivity. (**A-C**) Tumor growth in a xenograft model established by subcutaneously injecting LINC00665-knockdown A549 cells into NICD-SOD mice, with further intravenous injection of activated NK92 cells on days 3 and 7 post-implantation. (**D**) Ki-67 staining of tumor sections. (**E**) Flow cytometry analysis of CD107a + NK cells in the tumor. Data are presented as mean ± standard deviation. The comparison between the two groups was performed using an independent sample t-test. * indicates *P* < 0.05, ** indicates *P* < 0.01, *** indicates *P* < 0.001

To validate the in vitro findings, we established a xenograft mouse model. A549 cells with LINC00665 knockdown were subcutaneously injected into NOD-SCID mice, followed by intravenous injection of activated NK92 cells on days 3 and 7 post-implantation. As shown in Fig. 7A-C, LINC00665 knockdown significantly inhibited tumor growth, and NK cell injection further enhanced this effect (all *P* < 0.05). Ki-67 staining in xenograft tumors revealed a reduction in Ki-67-positive cells after LINC00665 knockdown, with a more pronounced decrease in the sh-LINC00665 + NK group (all *P* < 0.05, Fig. 7D). Additionally, flow cytometry further demonstrated that LINC00665 knockdown significantly increased the proportion of CD107a + NK cells in the xenograft tumors, and NK cell injection further augmented the number of NK cells (all *P* < 0.05, Fig. 7E). These results indicate that LINC00665 depletion suppresses tumor growth and enhances the sensitivity of NK cells in vivo.

## Discussion

LINC00665, as revealed by our analyses, exhibits elevated expression in both lung adenocarcinoma (LUAD) and lung squamous cell carcinoma (LUSC) patients, correlating with adverse prognosis. This finding aligns with the growing recognition of LINC00665 as a potential oncogenic player in various cancers. In particular, our investigation into its role in immune escape mechanisms uncovered a significant positive correlation with immune-suppressive cytokines (TGF-β, IL-10, and IL-1β) and a negative correlation with anti-tumor cytokines (IFN-γ, IL-2, and TNF-α) in metastatic lung cancer. These results strongly implicate LINC00665 in the orchestration of an immunosuppressive microenvironment favoring tumor progression. The impact of LINC00665 on lung cancer behavior was further elucidated through in vitro experiments. The observed downregulation of LINC00665 led to a substantial inhibition of cancer cell proliferation, migration, and invasion, coupled with an enhancement of apoptotic processes. These findings align with previous studies highlighting the role of LINC00665 in driving tumor aggressiveness, indicating its potential as a therapeutic target in lung cancer.

Lu et al. analyzed the expression level of LINC00665 in breast cancer tissues and matched normal tissues in the database [21]. They found that the expression of the LINC00665 was significantly upregulated in breast cancer tissues [21]. Furthermore, LINC00665 expression was significantly associated with the tumor size and TNM stage, but not with the age of the breast cancer patients [21]. In addition, Qi et al. conducted the Kaplan-Meier survival analysis to explore the correlation between LINC00665 expression and the prognosis of breast cancer patients [22]. They found that high expression of LINC00665 could predict poor overall survival of breast cancer patients [22]. Studies have reported that LINC00665 played a vital role in breast cancer progression, and LINC00665 Knockdown inhibited breast cancer cells proliferation, migration, and invasion but promoted apoptosis [21–23]. However, in lung cancer, Liu et al. [24] found that LINC00665 expression was significantly upregulated in lung cancer tissues and gefitinib-resistant cells. Moreover, they certified that LINC00665 could drive gefitinib resistance by increasing EZH2 and activating the PI3K/AKT pathway [24]. In addition, another study showed that LINC00665 interacted with YB-1 and promoted angiogenesis in lung cancer [25]. Furthermore, our study delved into the intricate crosstalk involving HHLA2, TCF7, and LINC00665. Notably, the correlation analysis from TCGA data pinpointed a significant positive association between LINC00665 and HHLA2, emphasizing their potential cooperative role in lung cancer progression. Mechanistically, LINC00665 was found to recruit the transcription factor TCF7, known for its involvement in cancer-related signaling pathways, to promote the transcription of HHLA2.

HHLA2 can provide a co-stimulatory signal to induce T cell activation or a co‐inhibitory signal to inhibit T cells, depending on the receptors it binds [26, 27]. Zhu et al. indicated that HHLA2 stimulated T cell proliferation and cytokine production via binding to TMIGD2 on antigen‐presenting cells [28]. However, Zhao et al. demonstrated that HHLA2 inhibited T cell proliferation and reduced cytokine production, thus to suppress T cell‐mediated antitumor responses [26]. Analysis of the gene expression chip GSE138682 using the R Corr package revealed that LINC00665 has the highest correlation with HHLA2. Human endogenous retrovirus-H long terminal repeat-associating protein 2 (HHLA2, also known as B7H5 and B7H7) is a member of the B7 family of immune regulatory ligands, and it is upregulated in various tumors. HHLA2 is upregulated in lung cancer cells, and its genetic deletion inhibits NSCLC growth and polarization of tumor-associated macrophages (TAM) towards M2 phenotype [29]. HHLA2 has been reported to be positively correlated with immune infiltration in liver cancer, and its upregulation induces M2 polarization and chemotaxis, contributing to immune escape and the development of hepatocellular carcinoma [30, 31]. Additionally, studies have reported that HHLA2 in chronic myeloid leukemia cells interacts with KIR3DL3 in NK cells (inhibitory receptor for HHLA2), leading to the inhibition of NK cell cytotoxicity [20]. Furthermore, analysis on the catRAPID website indicates that LINC00665 has binding sites targeting transcription factor TCF7. JASPAR website analysis shows that TCF7 has binding sites on the promoter of HHLA2, and LncATLAS database prediction suggests that LINC00665 is mainly localized in the cell nucleus. TCGA database queries show significantly high expression of TCF7 in LUAD. Based on these analyses, we hypothesize that LINC00665 may promote the transcription of HHLA2 in lung cancer cells by recruiting transcription factor TCF7, thereby inhibiting NK cell cytotoxicity and promoting immune escape and the development of lung cancer. However, there is currently no research reporting whether LINC00665 can promote the expression of HHLA2 in lung cancer cells and inhibit NK cell cytotoxicity by targeting binding TCF7, leading to immune escape and the development of lung cancer.

In conclusion, our study unravels a novel regulatory network involving LINC00665, TCF7, HHLA2, and immune escape in lung cancer. The identified molecular interactions provide a foundation for future therapeutic strategies aimed at disrupting immune-suppressive mechanisms and harnessing the anti-tumor potential of the immune system in lung cancer treatment.

### Electronic supplementary material

Below is the link to the electronic supplementary material.

Supplementary Material 1

Supplementary Material 2

Supplementary Material 3

## Data Availability

The datasets generated during and/or analysed during the current study are available from the corresponding author on reasonable request.
