# Supplementary material for: LINC00665 promotes the progression and immune evasion of lung cancer by facilitating the translation of TCF7 protein through dependence on IRES
Source: Cancer Cell Int. 2024 Jun 29;24:227. doi: 10.1186/s12935-024-03411-4 (PMC11218341; doi:10.1186/s12935-024-03411-4)
Supplement: Supplementary file 1 — Supplementary Material 1 [file 12935_2024_3411_MOESM1_ESM.docx]

**Fig S1**

**
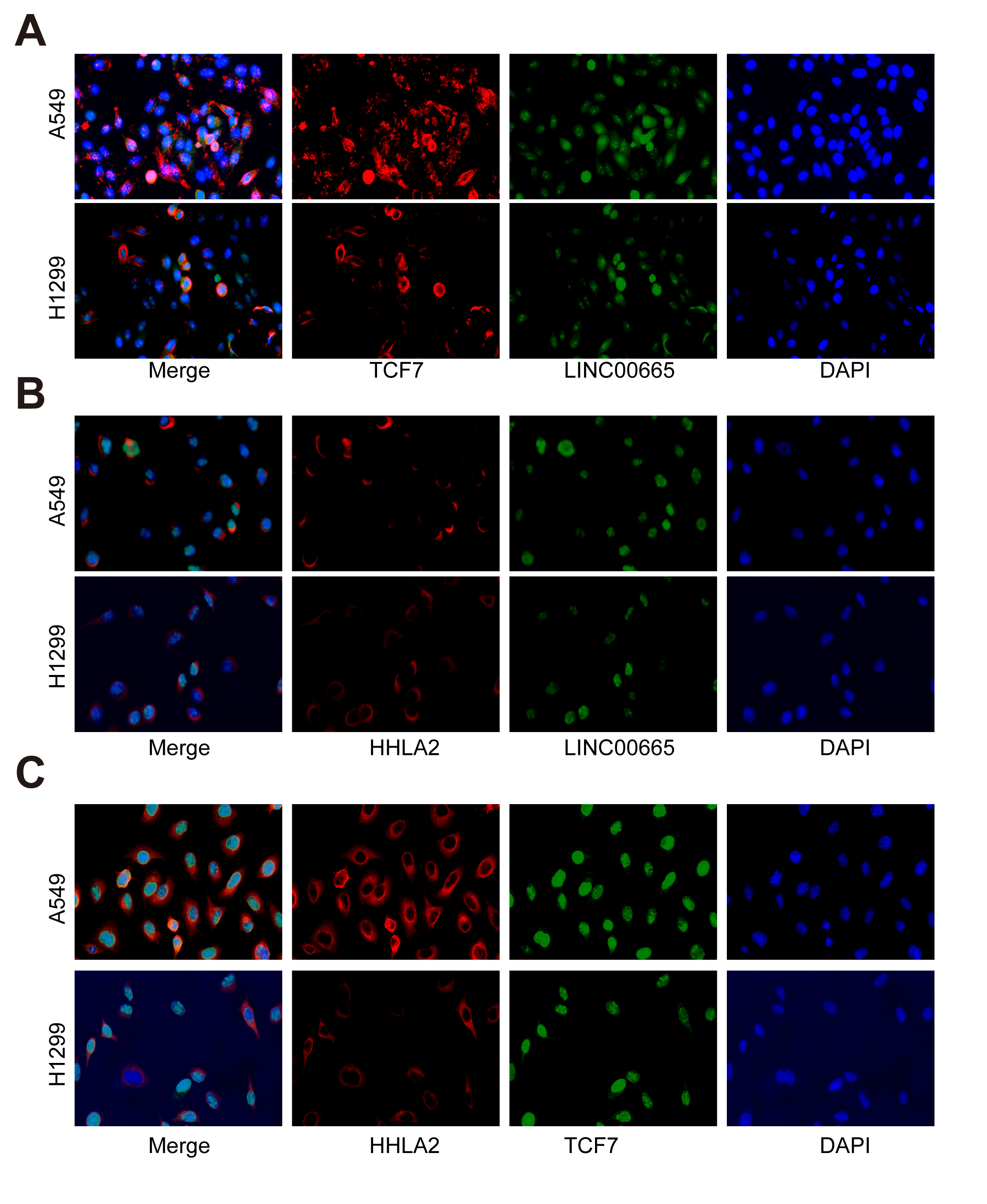
**

**Fig S1. The co-localization of LINC00665, TCF7 and HHLA2 in LC cells.** (A) The co-localization of LINC00665 and TCF7. (B) The co-localization of LINC00665 and HHLA2. (C) The co-localization of TCF7 and HHLA2.
