# Supplementary material for: LINC00665 promotes the progression and immune evasion of lung cancer by facilitating the translation of TCF7 protein through dependence on IRES
Source: Cancer Cell Int. 2024 Jun 29;24:227. doi: 10.1186/s12935-024-03411-4 (PMC11218341; doi:10.1186/s12935-024-03411-4)
Supplement: Supplementary file 2 — Supplementary Material 2 [file 12935_2024_3411_MOESM2_ESM.docx]

Supplementary Table 1. Characteristics of LC patients.

| Characteristics | N |
| --- | --- |
| Age |  |
| > 65 | 43 |
| < 65 | 41 |
| Lymph node metastasis |  |
| With | 26 |
| Without | 58 |
| Gender |  |
| Male | 52 |
| Female | 32 |
| BMI |  |
| > 25 | 39 |
| < 25 | 45 |
| Smoking status |  |
| Positive | 53 |
| Negative | 31 |
