# Supplementary figures and images for: LINC00665 promotes the progression and immune evasion of lung cancer by facilitating the translation of TCF7 protein through dependence on IRES
Source: Cancer Cell Int. 2024 Jun 29;24:227. doi: 10.1186/s12935-024-03411-4 (PMC11218341; doi:10.1186/s12935-024-03411-4)

4L

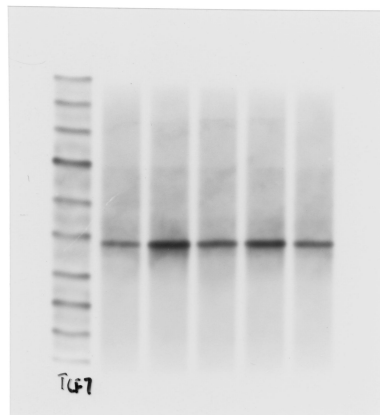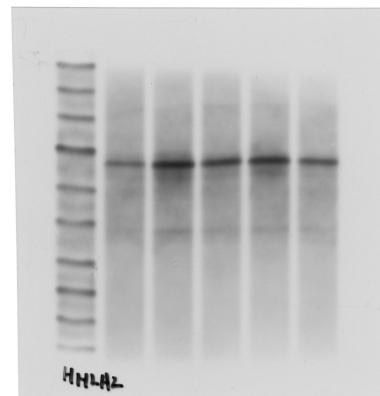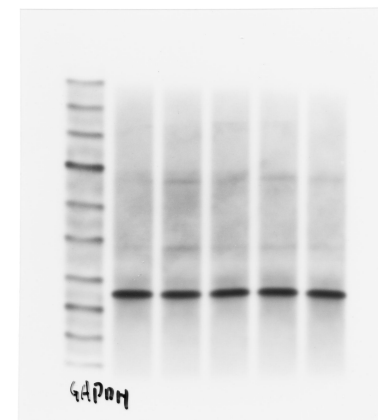

4N

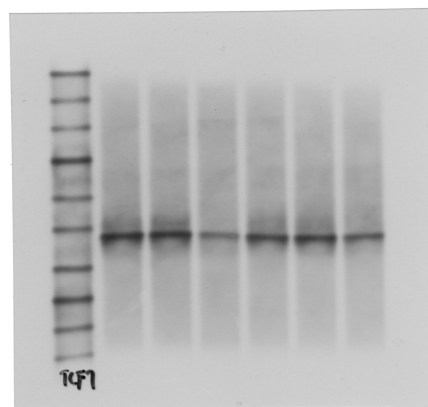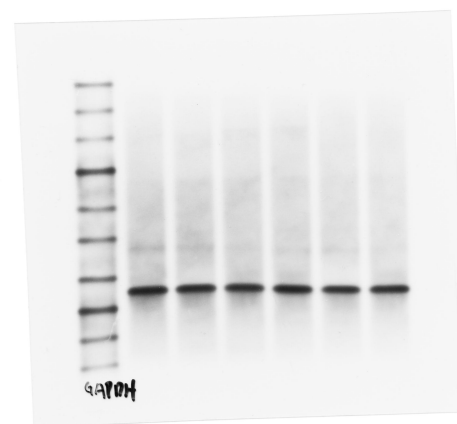

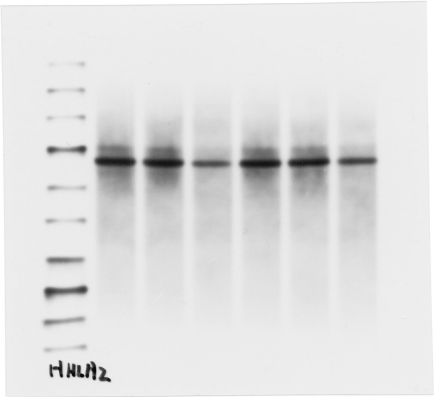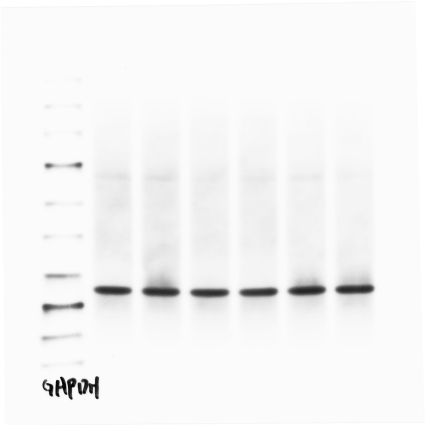

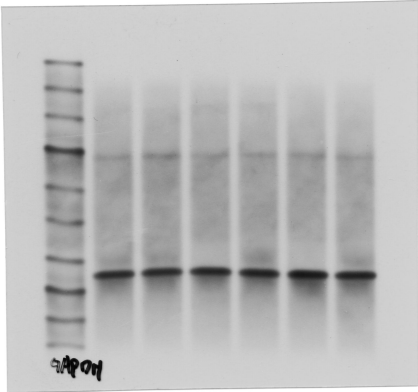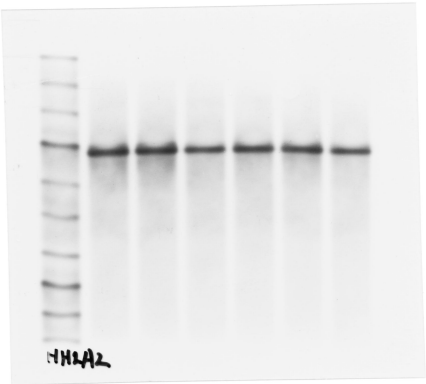

Supplement: Supplementary file 3 — Supplementary Material 3 [file 12935_2024_3411_MOESM3_ESM.pdf]
